# Liver tests and outcomes in heart failure with reduced ejection fraction: findings from DAPA‐HF

**DOI:** 10.1002/ejhf.2649

**Published:** 2022-08-22

**Authors:** Carly Adamson, Lorna M. Cowan, Rudolf A. de Boer, Mirta Diez, Jarosław Drożdż, Andre Dukát, Silvio E. Inzucchi, Lars Køber, Mikhail N. Kosiborod, Charlotta E.A. Ljungman, Felipe A. Martinez, Piotr Ponikowski, Marc S. Sabatine, Daniel Lindholm, Olof Bengtsson, David W. Boulton, Peter J. Greasley, Anna Maria Langkilde, Mikaela Sjöstrand, Scott D. Solomon, John J.V. McMurray, Pardeep S. Jhund

**Affiliations:** ^1^ BHF Cardiovascular Research Centre University of Glasgow Glasgow UK; ^2^ Department of Cardiology University Medical Center and University of Groningen Groningen The Netherlands; ^3^ Division of Cardiology Institute Cardiovascular de Buenos Aires Buenos Aires Argentina; ^4^ Department Cardiology Medical University of Lodz Lodz Poland; ^5^ Fifth Department of Internal Medicine Comenius University in Bratislava Bratislava Slovakia; ^6^ Section of Endocrinology Yale School of Medicine New Haven CT USA; ^7^ Department of Cardiology, Rigshospitalet Copenhagen University Hospital Copenhagen Denmark; ^8^ Saint Luke's Mid America Heart Institute University of Missouri Kansas City MO USA; ^9^ The George Institute for Global Health University of New South Wales Sydney NSW Australia; ^10^ Institute of Medicine, Department of Molecular and Clinical Medicine/Cardiology, Sahlgrenska Academy University of Gothenburg Gothenburg Sweden; ^11^ Universidad Nacional de Córdoba Córdoba Argentina; ^12^ Center for Heart Diseases, University Hospital Wroclaw Medical University Wroclaw Poland; ^13^ TIMI Study Group, Division of Cardiovascular Medicine Brigham and Women's Hospital Boston MA USA; ^14^ Late Stage Development, Cardiovascular, Renal and Metabolism, BioPharmaceuticals R&D AstraZeneca Gothenburg Sweden; ^15^ Clinical Pharmacology and Quantitative Pharmacology, Clinical Pharmacology and Safety Sciences, R&D AstraZeneca Gaithersburg MD USA; ^16^ Early Research and Development, Cardiovascular, Renal and Metabolism, BioPharmaceuticals R&D AstraZeneca Gothenburg Sweden; ^17^ Division of Cardiovascular Medicine Brigham and Women's Hospital Boston MA USA

**Keywords:** Heart failure, Dapagliflozin, SGLT2 inhibitor, Hepatic function, Bilirubin, Alkaline phosphatase

## Abstract

**Aims:**

Reflecting both increased venous pressure and reduced cardiac output, abnormal liver tests are common in patients with severe heart failure and are associated with adverse clinical outcomes. We aimed to investigate the prognostic significance of abnormal liver tests in ambulatory patients with heart failure with reduced ejection fraction (HFrEF), explore any treatment interaction between bilirubin and sodium–glucose cotransporter 2 (SGLT2) inhibitors and examine change in liver tests with SGLT2 inhibitor treatment.

**Methods and results:**

We explored these objectives in the Dapagliflozin And Prevention of Adverse outcomes in Heart Failure (DAPA‐HF) trial, with focus on bilirubin. We calculated the incidence of cardiovascular death or worsening heart failure by bilirubin tertile. Secondary cardiovascular outcomes were examined, along with the change in liver tests at the end‐of‐study visit. Baseline bilirubin was available in 4720 patients (99.5%). Participants in the highest bilirubin tertile (T3) have more severe HFrEF (lower left ventricular ejection fraction, higher N‐terminal pro‐B‐type natriuretic peptide [NT‐proBNP] and worse New York Heart Association class), had a greater burden of atrial fibrillation but less diabetes. Higher bilirubin (T3 vs. T1) was associated with worse outcomes even after adjustment for other predictive variables, including NT‐proBNP and troponin T (adjusted hazard ratio for the primary outcome 1.73 [95% confidence interval 1.37–2.17], *p* < 0.001; and 1.52 [1.12–2.07], *p* = 0.01 for cardiovascular death). Baseline bilirubin did not modify the benefits of dapagliflozin. During follow‐up, dapagliflozin had no effect on liver tests.

**Conclusion:**

Bilirubin concentration was an independent predictor of worse outcomes but did not modify the benefits of dapagliflozin in HFrEF. Dapagliflozin was not associated with change in liver tests.

Clinical Trial Registration: ClinicalTrials.gov NCT03036124.

## Graphical abstract

Summary of key findings in analysis of liver tests in DAPA‐HF. Using restricted cubic splines to model risk of the primary outcome by baseline bilirubin level as a continuous variable shows an essentially linear relationship, with higher bilirubin associated with increased risk. Forest plot for each outcome demonstrates the benefit of dapagliflozin is not modified by baseline bilirubin tertile. Bilirubin adds incremental information about risk of the primary outcome after adjustment for known prognostic markers (high‐sensitivity troponin T [hs‐TnT] and N‐terminal pro‐B‐type natriuretic peptide [NT‐proBNP]). CI, confidence interval; HR, hazard ratio.

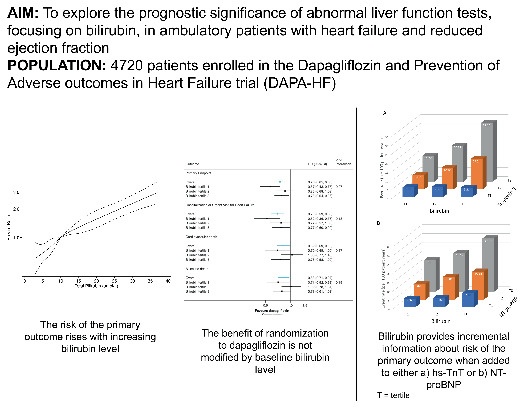

## Introduction

Liver tests are commonly abnormal in patients with severe heart failure (HF) and during acute decompensation.^1^, ^2^, ^3^, ^4^, ^5^, ^6^ In both settings, abnormal liver tests are predictive of worse subsequent outcomes, including death.^1^, ^2^, ^3^, ^4^, ^5^, ^6^ Abnormal liver tests are thought to reflect both increased venous pressure and reduced cardiac output.^7^, ^8^, ^9^

Much less is known about the prevalence or the prognostic significance of abnormal liver tests in ambulatory patients with HF, especially in such individuals receiving contemporary treatments.^10^, ^11^ In the Candesartan in Heart Failure: Assessment of Reduction in Mortality and Morbidity (CHARM) program, alkaline phosphatase (ALP) was elevated in 14.0% of patients, total bilirubin in 13.0%, alanine aminotransferase (ALT) in 3.1%, and aspartate aminotransferase (AST) in 4.1% of patients.^10^ In CHARM, bilirubin was the most powerful prognostic liver test and remained an independent predictor in a multivariable model, although that model did not include a natriuretic peptide. More recently, in the Prospective Comparison of ARNI (Angiotensin Receptor–Neprilysin Inhibitor) with ACEI (Angiotensin‐Converting Enzyme Inhibitor) to Determine Impact on Global Mortality and Morbidity in Heart Failure (PARADIGM‐HF) trial, 11.6% of patients with HF with reduced ejection fraction (HFrEF) were found to have elevated bilirubin at baseline and bilirubin was again the most predictive liver test and remained independently so in a multivariable model including N‐terminal pro‐B‐type natriuretic peptide (NT‐proBNP).^11^ Whether bilirubin remains predictive when additional prognostic biomarkers such as high‐sensitivity troponin T are included is not known.^12^

Recently sodium–glucose cotransporter 2 (SGLT2) inhibitors have been introduced as a treatment for the treatment of HFrEF.^13^, ^14^ Due to their effect on proximal renal tubular reabsorption of glucose, coupled with sodium, these agents cause an initial osmotic diuresis and natriuresis which might relieve hepatic congestion and improve liver tests.^15^, ^16^, ^17^ It has also been suggested that SGLT2 inhibitors might reduce liver fat in patients with type 2 diabetes, a condition linked to obesity and frequently associated with non‐alcoholic fatty liver disease.^18^, ^19^, ^20^

We have investigated the prevalence and predictive importance of abnormal liver tests in the Dapagliflozin And Prevention of Adverse outcomes in Heart Failure (DAPA‐HF) trial and the effect of dapagliflozin on liver tests in this trial.^13^, ^21^

## Methods

DAPA‐HF was a randomized, double‐blind, placebo‐controlled trial in patients with HFrEF, which evaluated the efficacy and safety of dapagliflozin 10 mg once daily, added to standard care.^13^, ^21^ Ethics Committees at each of the 410 participating institutions in 20 countries approved the protocol, all patients provided written informed consent and the study complied with the Declaration of Helsinki.

### Study patients

Men and women aged ≥18 years, in New York Heart Association (NYHA) functional class II–IV, with a left ventricular ejection fraction (LVEF) ≤40%, and an elevated NT‐proBNP level, were eligible provided they were receiving optimal pharmacological and device therapy in the opinion of the investigator.^13^, ^21^ The main exclusion criteria included type 1 diabetes mellitus, symptomatic hypotension, systolic blood pressure <95 mmHg and estimated glomerular filtration rate (eGFR) <30 ml/min/1.73 m^2^. Patients with an AST or ALT more than three times the upper limit of normal, or total bilirubin more than two times the upper limit of normal were also excluded, as were patients judged to have a life expectancy of <2 years due to a condition other than HF.^13^, ^21^

### Measurement of liver tests

Alkaline phosphatase, ALT, AST, and total bilirubin were measured at enrolment and at the end‐of‐study visit. Samples were processed in a central laboratory. Our main analysis of change in liver tests used the end‐of‐study measurement, which could fall at 12, 16, 20, or 24 months. Although not required, some patients had measurements taken at other scheduled visits, at the investigator's discretion, or at unscheduled visits. In an exploratory analysis, we included the results from this unplanned sampling, allocating them to the nearest scheduled visit.

### Pre‐specified trial outcomes

The primary outcome of DAPA‐HF was the composite of worsening HF (HF hospitalization or urgent visit for HF requiring intravenous therapy) or cardiovascular (CV) death, whichever occurred first. Pre‐specified secondary endpoints included HF hospitalization or CV death; and HF hospitalizations (first and recurrent) and CV deaths. The change from baseline to 8 months in Kansas City Cardiomyopathy Questionnaire total symptom score (KCCQ‐TSS) was an additional secondary endpoint, with the proportion having a 5‐point or more increase or decrease in their score at 8 months determined as previously described.^13^, ^21^ There was also a pre‐specified secondary renal composite outcome, but this was not evaluated further in this study because of the small number of events.

### Definition of elevated liver tests

The upper limits of normal were 35 IU/L for AST and ALT, 1.0 mg/dl for bilirubin, and 120 IU/L for ALP.^22^ Given the evidence that bilirubin is the most prognostically important liver test, this was the focus of our analysis of the association with subsequent clinical outcomes, as described in the statistical analysis section below.

### Statistical analysis

Patients were grouped by baseline bilirubin measurement into tertiles and baseline characteristics were summarized as means (standard deviations), median (interquartile ranges [IQR]), or percentages. Logistic regression was used to explore associations with elevated bilirubin at baseline, examining candidate variables in a univariable model and those with a *p*‐value <0.2 being added into a stepwise logistic regression model.

Kaplan–Meier estimates and Cox proportional‐hazards models, stratified by diabetes status, and adjusted for treatment‐group assignment and history of HF hospitalization (except for all‐cause death) were used to examine the primary and secondary outcomes across bilirubin tertiles, with further models adjusted for known predictors of risk of HF endpoints (age, sex, race, region, systolic blood pressure, heart rate, LVEF, eGFR, NT‐proBNP [log‐transformed], NYHA class, hypertension, previous stroke, previous myocardial infarction, atrial fibrillation, and HF aetiology). A second adjusted model included the same listed variables and the addition of high‐sensitivity troponin T (log‐transformed). A semi‐parametric proportional‐rates model was used to evaluate recurrent HF hospitalizations and CV death.^23^

Each liver test was considered as a continuous variable in Cox regression models for the same outcomes and adjustments after being log‐transformed to normalize distribution. The relationship between continuous liver tests and each outcome was further explored using restricted cubic splines to examine for a non‐linear relationship.

As NT‐proBNP and troponin T are established powerful predictors of outcome in HF, the incremental predictive value of bilirubin added to these biomarkers was examined. Rates of the primary outcome were assessed in groups defined by tertile of both bilirubin and either NT‐proBNP or troponin T to evaluate the effect of elevation of both markers on the occurrence of the primary outcome. Groups defined by combinations of tertiles of both biomarkers were compared in a Cox model.

The effects of randomized treatment on outcomes within each tertile of bilirubin was evaluated and modification of treatment effects by baseline bilirubin tertile was assessed using a global interaction test. The differences between treatment groups in the proportion of patients with a clinically significant (≥5 points) improvement or deterioration in KCCQ‐TSS at 8 months was analysed using the methods described previously and presented as an odds ratio for each baseline bilirubin category.^13^, ^21^ The effect of dapagliflozin compared with placebo on each endpoint was examined across the range of baseline bilirubin as a continuous variable using restricted cubic splines. This was repeated for the other liver tests for the primary endpoint.

Change in liver tests was analysed using a least‐square means regression and by the ratio of geometric means between baseline and end‐of‐study visit. To use additional unplanned samples, recordings at 12 or 16 months of follow‐up were combined (either a recording at 12 or 16 months or if both present the mean of the two recorded values) as well as 20‐ and 24‐month follow‐up and analysed in the same manner.

Safety analyses were performed in randomized patients who had received at least one dose of dapagliflozin or placebo. The interaction between baseline bilirubin tertile and randomized treatment on the occurrence of the pre‐specified safety outcomes was tested in a logistic regression model.

All analyses were conducted using Stata version 17.0 (StataCorp, College Station, TX, USA) and SAS version 9.4 (SAS Institute, Cary, NC, USA). A *p*‐value <0.05 was considered statistically significant.

## Results

A baseline measurement of bilirubin was available in 4720 patients (99.5%) and showed a right‐skewed distribution (online supplementary *Figure* *S1*); ALP was available in 4729 patients, ALT in 4714, and AST in 4681. Baseline median (IQR) values were: total bilirubin 10 (7–14) μmol/L (1 mg/dl = 17.1 μmol/L for conversion); ALP 76 (62–96) IU/L; ALT 18 (13–24) IU/L; and AST 21 (17–26) IU/L. The proportion of patients with a level above the upper limit of normal was: 661 (14.0%) for bilirubin; 471 (10.0%) for ALP; 393 (8.3%) for ALT; and 368 (7.9%) for AST. A total of 537 patients had either elevated AST or ALT or both, and in 86 patients this was accompanied by a raised bilirubin.

### Baseline characteristics

There were many significant differences according to baseline bilirubin level (*Table* *1*). Each of ALP, ALT and AST were higher in participants with higher bilirubin. Participants with higher bilirubin were more likely to be male (T3 85.3% vs. T1 67.6%), to have lower systolic blood pressure (120.0 ± 15.6 vs. 123.8 ± 16.9 mmHg), lower LVEF (30.5 ± 7.0% vs. 31.7 ± 6.4%), and higher NT‐proBNP (1815 [1044–3406] vs. 1217 [741–2220] pg/ml) (all *p* < 0.001). Patients with higher bilirubin were more likely to be in NYHA class III/IV (than class II) and to have a lower (worse) KCCQ‐TSS than those in the lowest bilirubin tertile (*Table* *1*). Other differences between patients in bilirubin T3 versus T1 included a lower prevalence of diabetes (42.4% vs. 48.1%) but a substantially higher prevalence of atrial fibrillation (49.1% vs. 27.9% by medical history, 34.3% vs. 14.5% with baseline electrocardiogram in atrial fibrillation or flutter) (*Table* *1*). Patients in the highest bilirubin tertile were more likely than those in the lowest tertile to be treated with digoxin, an oral anticoagulant or a mineralocorticoid receptor antagonist, and less often treated with an antiplatelet agent and statin. Among patients with diabetes, fewer in the highest bilirubin tertile were prescribed treatments for diabetes, although glycated haemoglobin was similar across baseline bilirubin tertiles.

**Table 1:** Baseline characteristics according to tertile of total bilirubin at baseline

	Bilirubin range (μmol/L)			*p*‐value (trend)
	Tertile 1 (3–8) (*n* = 1612)	Tertile 2 (8.6–12) (*n* = 1602)	Tertile 3 (13–63) (*n* = 1506)	
Age (years)	66.0 ± 10.6	66.9 ± 10.7	66.2 ± 11.3	0.145
Female sex, *n* (%)	522 (32.4)	361 (22.5)	222 (14.7)	<0.001
BMI (kg/m^2^)	28.5 ± 6.5	28.0 ± 5.7	28.0 ± 5.6	0.270
BMI categories				<0.001
Underweight and normal weight	468 (29.0)	475 (29.7)	398 (26.4)	
Overweight	552 (34.2)	569 (35.6)	592 (39.3)	
Obesity class I	324 (20.1)	356 (22.2)	328 (21.8)	
Obesity class II–III	268 (16.6)	200 (12.5)	188 (12.5)	
Race (%)				0.025
White	1132 (70.2)	1125 (70.2)	1059 (70.3)	
Black	97 (6.0)	76 (4.7)	52 (3.5)	
Asian	356 (22.1)	378 (23.6)	376 (25.0)	
Other	27 (1.7)	23 (1.4)	19 (1.3)	
Region (%)				<0.001
North America	232 (14.4)	270 (16.9)	169 (11.2)	
South America	312 (19.4)	296 (18.5)	203 (13.5)	
Europe	718 (44.5)	668 (41.7)	761 (50.5)	
Asia/Pacific	350 (21.7)	368 (23.0)	373 (24.8)	
SBP (mmHg)	123.8 ± 16.9	121.5 ± 16.2	120.0 ± 15.6	<0.001
Pulse pressure (mmHg)	50.6 ± 13.3	48.4 ± 12.3	45.9 ± 11.8	<0.001
HR (bpm)	71.2 ± 11.1	71.0 ± 11.6	72.4 ± 12.4	0.025
Alkaline phosphatase (IU/L)	75.0 [62.0–92.0]	76.0 [62.0–95.0]	78.0 [63.0–103.0]	<0.001
Aspartate transaminase (IU/L)	19.0 [16.0–24.0]	21.0 [17.0–26.0]	22.0 [18.0–28.0]	<0.001
Alanine transaminase (IU/L)	17.0 [13.0–23.0]	18.0 [13.0–25.0]	18.0 [14.0–25.0]	<0.001
eGFR (ml/min/1.73 m^2^)	65.1 ± 20.2	65.3 ± 19.0	66.9 ± 18.8	0.003
eGFR <60 ml/min/1.73 m^2^, *n* (%)	702 (43.5)	641 (40.0)	573 (38.0)	0.002
NT‐proBNP (pg/ml)	1217.0 [740.9–2220.4]	1423.6 [862.8–2413.7]	1814.8 [1044.1–3406.2]	<0.001
NT‐proBNP if baseline ECG AF/flutter (pg/ml)	1879.8 [1245.8–2974.7]	1826.4 [1220.0–2834.0]	2165.8 [1296.0–3490.4]	0.009
NT‐proBNP if baseline ECG not AF/flutter (pg/ml)	1112.8 [687.0–2025.1]	1300.9 [779.0–2294.2]	1619.6 [906.0–3360.2]	<0.001
Baseline blood urea nitrogen (mg/dl)	21.3 [16.7–27.7]	21.0 [17.0–26.9]	21.0 [17.0–27.2]	0.857
HbA1c (%)	6.2 [5.7–7.0]	6.0 [5.7–6.8]	6.1 [5.7–6.7]	0.003
HbA1c in patients without diabetes (%)	5.8 [5.5–6.0]	5.7 [5.5–6.0]	5.8 [5.5–6.0]	0.870
HbA1c in patients with diabetes (%)	7.1 [6.5–8.1]	6.9 [6.4–8.0]	6.9 [6.4–7.8]	0.031
Urate (mg/dl)	6.0 ± 1.7	6.1 ± 1.7	6.3 ± 1.7	<0.001
Haemoglobin (g/dl)	13.0 ± 1.5	13.6 ± 1.6	14.0 ± 1.6	<0.001
Haematocrit (%)	40.1 ± 4.7	41.6 ± 4.8	42.8 ± 5.2	<0.001
Ischaemic aetiology, *n* (%)	920 (57.1)	911 (56.9)	830 (55.1)	0.275
HF diagnosis duration, *n* (%)				0.211
0–3 months	52 (3.2)	49 (3.1)	46 (3.1)	
>3–6 months	146 (9.1)	124 (7.7)	118 (7.8)	
>6–12 months	209 (13.0)	186 (11.6)	160 (10.6)	
>1–2 years	251 (15.6)	212 (13.2)	219 (14.5)	
>2–5 years	360 (22.3)	385 (24.0)	357 (23.7)	
>5 years	594 (36.8)	646 (40.3)	606 (40.2)	
Time from last HF hospitalization to randomization, *n* (%)				0.377
0–3 months	116 (7.2)	117 (7.3)	130 (8.6)	
>3–6 months	139 (8.6)	145 (9.1)	124 (8.2)	
>6–12 months	196 (12.2)	167 (10.4)	159 (10.6)	
>1–2 years	127 (7.9)	114 (7.1)	106 (7.0)	
>2–5 years	118 (7.3)	124 (7.7)	92 (6.1)	
>5 years	82 (5.1)	101 (6.3)	83 (5.5)	
No prior HF hospitalization, *n* (%)	834 (51.7)	834 (52.1)	812 (53.9)	
Ejection fraction (%)	31.7 ± 6.4	30.9 ± 6.9	30.5 ± 7.0	<0.001
NYHA class III/IV, *n* (%)	459 (28.5)	494 (30.8)	581 (38.6)	<0.001
Total KCCQ score at baseline	79.2 [58.3–93.8]	79.2 [60.4–91.7]	75.5 [57.3–91.7]	0.013
Hypertension, *n* (%)	1195 (74.1)	1205 (75.2)	1109 (73.6)	0.768
Type 2 diabetes, *n* (%)	776 (48.1)	714 (44.6)	638 (42.4)	0.001
Duration of type 2 diabetes, *n* (%)				0.007
<1 year	89 (12.1)	83 (12.6)	80 (13.9)	
1–5 years	151 (20.5)	157 (23.8)	155 (26.9)	
5–10 years	160 (21.7)	162 (24.5)	139 (24.1)	
>10 years	338 (45.8)	258 (39.1)	202 (35.1)	
History of atrial fibrillation, *n* (%)	449 (27.9)	624 (39.0)	739 (49.1)	<0.001
Baseline ECG AF or flutter, *n* (%)	233 (14.5)	376 (23.5)	516 (34.3)	<0.001
Hospitalization for HF, *n* (%)	778 (48.3)	768 (47.9)	694 (46.1)	0.227
Previous myocardial infarction, *n* (%)	709 (44.0)	726 (45.3)	650 (43.2)	0.661
Previous stroke, *n* (%)	155 (9.6)	167 (10.4)	143 (9.5)	0.925
Chronic obstructive pulmonary disease, *n* (%)	209 (13.0)	212 (13.2)	158 (10.5)	0.038
Smoking status, *n* (%)				0.026
Current	267 (16.6)	234 (14.6)	191 (12.7)	
Former	696 (43.2)	724 (45.2)	663 (44.0)	
Never	649 (40.3)	644 (40.2)	652 (43.3)	
ACEi/ARB/ARNI, *n* (%)	1527 (94.7)	1494 (93.3)	1397 (92.8)	0.024
Diuretic, *n* (%)	1498 (92.9)	1492 (93.1)	1423 (94.5)	0.080
Digoxin, *n* (%)	256 (15.9)	288 (18.0)	341 (22.6)	<0.001
Beta‐blocker, *n* (%)	1549 (96.1)	1550 (96.8)	1436 (95.4)	0.303
Mineralocorticoid receptor antagonist, *n* (%)	1114 (69.1)	1128 (70.4)	1114 (74.0)	0.003
Oral anticoagulant, *n* (%)	511 (31.7)	696 (43.4)	754 (50.1)	<0.001
Antiplatelet, *n* (%)	965 (59.9)	885 (55.2)	730 (48.5)	<0.001
Statin, *n* (%)	1098 (68.1)	1103 (68.9)	960 (63.7)	0.011
Amiodarone, *n* (%)	218 (13.5)	275 (17.2)	234 (15.5)	0.108
ICD/CRT‐D, *n* (%)	400 (24.8)	450 (28.1)	386 (25.6)	0.575
Diabetes treatments in patients with T2DM, *n* (%)	*n* = 776	*n* = 714	*n* = 638	
Biguanide	404 (52.1)	336 (47.1)	272 (42.6)	<0.001
Sulfonylurea	181 (23.3)	143 (20.0)	109 (17.1)	0.004
DPP‐4 inhibitor	130 (16.8)	117 (16.4)	62 (9.7)	<0.001
GLP‐1 receptor agonist	12 (1.5)	4 (0.6)	4 (0.6)	0.064
Insulin	215 (27.7)	187 (26.2)	137 (21.5)	0.008

Continuous variables are expressed as mean ± standard deviation, or median [interquartile range], as appropriate.

ACEi, angiotensin‐converting enzyme inhibitor; AF, atrial fibrillation; ARB, angiotensin receptor blocker; ARNI, angiotensin receptor–neprilysin inhibitor; BMI, body mass index; BP, blood pressure; COPD, chronic obstructive pulmonary disease; CRT‐D, cardiac resynchronization therapy‐defibrillator; DPP‐4, dipeptidyl peptidase‐4; ECG, electrocardiogram; eGFR, estimated glomerular filtration rate; GLP‐1, glucagon‐like peptide‐1; HbA1c, glycated haemoglobin; HF, heart failure; HR, heart rate; ICD, implantable cardioverter‐defibrillator; KCCQ, Kansas City Cardiomyopathy Questionnaire; NT‐proBNP, N‐terminal pro‐B‐type natriuretic peptide; NYHA, New York Heart Association; SBP, systolic blood pressure; T2DM, type 2 diabetes mellitus.

A *p*‐value for trend across tertiles of bilirubin is reported, using the Cochran–Armitage test for binary response variables and the Jonckheere–Terpstra test for continuous variables. Multiple level categorical variables were compared using a chi‐squared test.

For conversion of bilirubin units, 1 mg/dl = 17.1 μmol/L.

The baseline characteristics identified through stepwise logistic regression independently associated with bilirubin are shown in online supplementary *Table* *S1*. Higher NT‐proBNP, higher haemoglobin, atrial fibrillation, higher (worse) NYHA class, male sex, lower pulse pressure, higher ALP, higher AST and lower KCCQ‐TSS score were associated with bilirubin above the normal range at baseline.

### Cardiovascular outcomes according to baseline bilirubin

#### Primary and secondary trial outcomes related to bilirubin level

**Figure 1:**
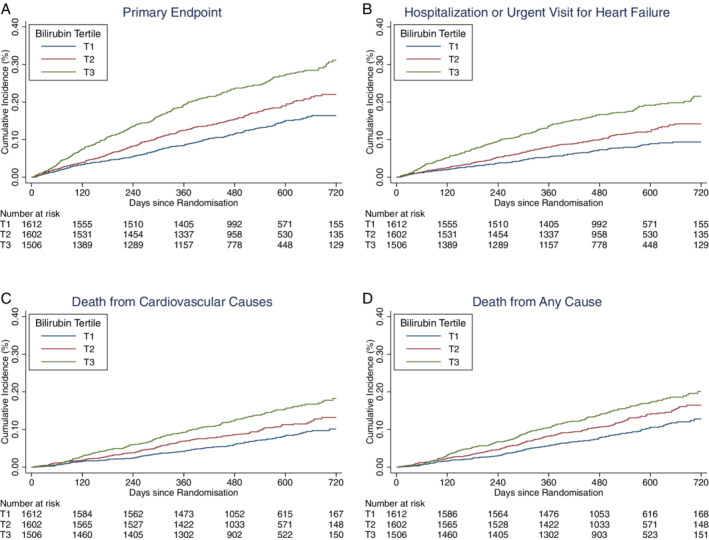
Unadjusted estimates for the cumulative incidence of the main study endpoints in tertiles of baseline bilirubin. Kaplan–Meier curves for each tertile of baseline bilirubin for the primary composite endpoint (*A*), hospitalization or urgent visit for heart failure (*B*), death from cardiovascular causes (*C*), or death from any cause (*D*).

Incidence rates for the primary and secondary outcomes of the trial were substantially higher in patients in bilirubin T3, compared to T1 (*Table* *2*, *Figure* *1*). The elevated risk associated with higher bilirubin persisted after comprehensive adjustment for other predictors of worse outcomes, including LVEF, NT‐proBNP and troponin T, with a fully adjusted hazard ratio (aHR) in bilirubin T3 versus T1 for the primary outcome of 1.73 (95% confidence interval [CI] 1.37–2.17, *p* < 0.001). The aHR for CV death (T3 vs. T1) was 1.52 (1.12–2.07; *p* = 0.01). Given more patients in the highest bilirubin tertile were male, this analysis was repeated in male patients only, with consistent results (online supplementary *Table* *S2*).

**Table 2:** Hazard ratios/rate ratios for key study outcomes according to tertile of total bilirubin at baseline

	Tertile 1 (*n* = 1612)	Tertile 2 (*n* = 1602)	*p*‐value	Tertile 3 (*n* = 1506)	*p*‐value
Primary endpoint, *n* (%)	215 (13.3)	287 (17.9)		383 (25.4)	
Event rate, no. of cases per 100 patient‐years (95% CI)	9.3 (8.1–10.6)	12.8 (11.4–14.4)		19.4 (17.6–21.5)	
Unadjusted hazard ratio (95% CI)	1.00 (reference)	1.41 (1.18–1.68)	<0.001	2.17 (1.83–2.56)	<0.001
Adjusted hazard ratio 1 (95% CI)	1.00 (reference)	1.23 (1.02–1.47)	0.03	1.66 (1.38–1.99)	<0.001
Adjusted hazard ratio 2 (95% CI)	1.00 (reference)	1.25 (0.99–1.58)	0.06	1.73 (1.37–2.17)	<0.001
HF urgent visit or hospitalization, *n* (%)	125 (7.8)	180 (11.2)		256 (17.0)	
Event rate, no. of cases per 100 patient‐years (95% CI)	5.4 (4.5–6.4)	8.0 (6.9–9.3)		13.0 (11.5–14.7)	
Unadjusted hazard ratio (95% CI)	1.00 (reference)	1.53 (1.22–1.92)	<0.001	2.51 (2.02–3.11)	<0.001
Adjusted hazard ratio 1 (95% CI)	1.00 (reference)	1.32 (1.04–1.67)	0.02	1.95 (0.54–2.45)	<0.001
Adjusted hazard ratio 2 (95% CI)	1.00 (reference)	1.39 (1.03–1.87)	0.03	2.10 (1.57–2.82)	<0.001
Death from cardiovascular causes, *n* (%)	120 (7.4)	165 (10.3)		214 (14.2)	
Event rate, no. of cases per 100 patient‐years (95% CI)	5.0 (4.2–6.0)	7.0 (6.0–8.2)		10.0 (8.7–11.4)	
Unadjusted hazard ratio (95% CI)	1.00 (reference)	1.43 (1.13–1.81)	0.003	2.06 (1.64–2.57)	<0.001
Adjusted hazard ratio 1 (95% CI)	1.00 (reference)	1.25 (1.98–1.60)	0.07	1.47 (1.15–1.88)	0.002
Adjusted hazard ratio 2 (95% CI)	1.00 (reference)	1.33 (0.98–1.81)	0.07	1.52 (1.12–2.07)	0.01
Death from any cause, *n* (%)	154 (9.6)	208 (13.0)		242 (16.1)	
Event rate, no. of cases per 100 patient‐years (95% CI)	6.4 (5.5–7.5)	8.8 (7.7–10.1)		11.3 (9.9–12.8)	
Unadjusted hazard ratio (95% CI)	1.00 (reference)	1.41 (1.14–1.73)	0.001	1.81 (1.47–2.21)	<0.001
Adjusted hazard ratio 1 (95% CI)	1.00 (reference)	1.24 (1.00–1.54)	0.05	1.35 (1.08–1.69)	0.01
Adjusted hazard ratio 2 (95% CI)	1.00 (reference)	1.30 (0.99–1.71)	0.06	1.37 (1.04–1.81)	0.03
Total HF hospitalizations and cardiovascular deaths, *n*	297	423		584	
Event rate, no. of events per 100 patient‐years (95% CI)	12.4 (11.0–13.9)	18.0 (16.4–19.8)		27.3 (25.2–29.6)	
Unadjusted rate ratio (95% CI)	1.00 (reference)	1.49 (1.29–1.73)	<0.001	2.30 (2.00–2.65)	<0.001
Adjusted hazard ratio 1 (95% CI)	1.00 (reference)	1.30 (1.11–1.51)	0.001	1.72 (1.48–2.00)	<0.001
Adjusted hazard ratio 2 (95% CI)	1.00 (reference)	1.42 (1.17–1.72)	<0.001	1.85 (1.53–2.24)	<0.001

CI, confidence interval; HF, heart failure.

For the primary endpoint, HF urgent visit or hospitalization, cardiovascular death, and death from any cause, models are adjusted for previous hospitalization for HF and treatment allocation and stratified by diabetic status. For death from any cause, models are adjusted for treatment allocation and stratified according to diabetic status. Adjusted model 1 includes additional adjustment for age, sex, race, region, systolic blood pressure, heart rate, left ventricular ejection fraction, estimated glomerular filtration rate, N‐terminal pro‐B‐type natriuretic peptide (log‐transformed), haemoglobin, New York Heart Association class, hypertension, previous stroke, previous myocardial infarction, atrial fibrillation, and HF aetiology. Adjusted model 2 has the same variables as model 1 with plus high‐sensitivity troponin T (log transformed).

**Figure 2:**
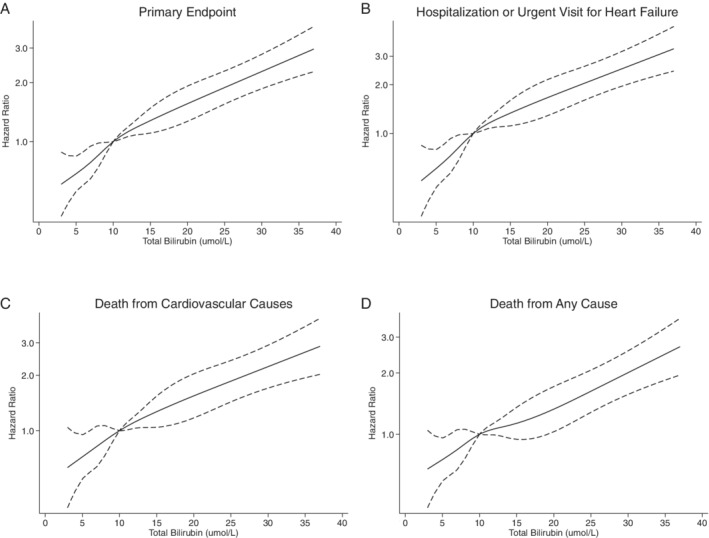
Association between total bilirubin as a continuous variable and the risk of the main study endpoints. Restricted cubic splines for the risk of the primary composite endpoint (*A*), hospitalization or urgent visit for heart failure (*B*), death from cardiovascular causes (*C*), or death from any cause (*D*) with the median bilirubin value (10 μmol/L) as reference.

Analyses using baseline bilirubin concentration as a continuous variable showed an essentially linear relationship between event rates and bilirubin level (*Figure* *2*). For each unit increase in log‐transformed total bilirubin, in adjusted Cox models, the aHR for the primary endpoint was 1.66 (1.39–1.98; *p* < 0.001); for hospitalization or urgent visit for HF 1.94 (1.56–2.40; *p* < 0.001); for death from CV causes 1.46 (1.16–1.85; *p* = 0.001); and for death from any cause 1.33 (1.07–1.64; *p* = 0.01) (online supplementary *Table* *S3*).

Analysed as a continuous variable, higher levels of ALP were associated with a higher risk of the primary outcome, components of the primary outcome, death from CV cause and any cause and recurrent HF hospitalization and CV death. In the adjusted model including NT‐proBNP and high‐sensitivity troponin T, the relationship remained significant for death from any cause only. Neither AST nor ALT level was associated with the risk of any outcome in either adjusted or unadjusted models (online supplementary *Table* *S3*).

Modelling the relationship between liver tests and risk of each outcome flexibly using restricted cubic splines showed an essentially linear relationship between elevated ALP and risk of outcomes, with some plateauing at very high levels of ALP (online supplementary *Figure* *S2*). The lack of relationship between AST and ALT and any outcome was confirmed.

Allocating patients to groups defined by tertiles of bilirubin and troponin T showed a marked increase in the rate of the primary outcome when both bilirubin and troponin T were in the highest tertile (*Graphical Abstract*). A similar outcome was seen with tertiles of bilirubin and NT‐proBNP (*Graphical Abstract*).

#### Effect of dapagliflozin on primary and secondary trial outcomes

The efficacy of dapagliflozin in preventing the primary outcome of CV death or worsening HF did not differ across bilirubin tertiles (*p* for interaction = 0.07). The efficacy of dapagliflozin in preventing CV death, worsening HF events and all‐cause death also did not differ by bilirubin tertile (*Table* *3*, *Graphical Abstract*). The results were similar when bilirubin was treated as a continuous variable (online supplementary *Figure* *S3*).

**Table 3:** Effect of dapagliflozin compared with placebo according to tertile of total bilirubin at baseline

	Tertile 1 (*n* = 1612)		Tertile 2 (*n* = 1602)		Tertile 3 (*n* = 1506)		*p*‐value for interaction
	Dapagliflozin (*n* = 818)	Placebo (*n* = 794)	Dapagliflozin (*n* = 798)	Placebo (*n* = 804)	Dapagliflozin (*n* = 741)	Placebo (*n* = 765)	
Primary endpoint							
Total events, *n* (%)	82 (10.0)	133 (16.8)	134 (16.8)	153 (19.0)	169 (22.8)	214 (28.0)	
Rate per 100 patient‐years (95% CI)	6.8 (5.5–8.4)	11.9 (10.1–14.2)	12.0 (10.1–14.2)	13.7 (11.7–16.0)	17.1 (14.7–19.9)	21.8 (19.1–24.9)	
Unadjusted HR	0.57 (0.43–0.75)		0.86 (0.68–1.09)		0.78 (0.64–0.96)		0.07
Adjusted HR^a^	0.55 (0.42–0.73)		0.86 (0.68–1.09)		0.79 (0.64–0.97)		
Hospitalization for HF or urgent HF visit							
Total events, *n* (%)	45 (5.5)	80 (10.1)	79 (9.9)	101 (12.6)	112 (15.1)	144 (18.8)	
Rate per 100 patient‐years	3.7 (2.8–5.0)	7.2 (5.8–8.9)	7.0 (5.7–8.8)	9.0 (7.4–11.0)	11.3 (9.4–13.6)	14.7 (12.5–17.3)	
Unadjusted HR	0.52 (0.36–0.75)		0.77 (0.57–1.03)		0.77 (0.60–0.99)		0.19
Adjusted HR^a^	0.51 (0.35–0.73)		0.76 (0.56–1.03)		0.77 (0.60–0.99)		
CV death							
*n* (%)	51 (6.2)	69 (8.7)	84 (10.5)	81 (10.1)	92 (12.4)	122 (16.0)	
Rate per 100 patient‐years (95% CI)	4.1 (3.1–5.4)	5.9 (4.6–7.4)	7.2 (5.8–8.9)	6.8 (5.5–8.5)	8.6 (7.0–10.6)	11.3 (9.4–13.4)	
Unadjusted HR	0.70 (0.49–1.00)		1.05 (0.77–1.43)		0.76 (0.58–1.00)		0.17
Adjusted HR^a^	0.67 (0.46–0.97)		1.03 (0.75–1.40)		0.82 (0.62–1.08)		
All‐cause death							
*n* (%)	66 (8.1)	88 (11.1)	104 (13.0)	104 (12.9)	106 (14.3)	136 (17.8)	
Rate per 100 patient‐years (95% CI)	5.3 (4.2–6.8)	7.5 (6.1–9.2)	8.9 (7.4–10.8)	8.8 (7.2–10.6)	9.9 (8.2–12.0)	12.5 (10.6–14.8)	
Unadjusted HR	0.71 (0.52–0.98)		1.02 (0.78–1.34)		0.79 (0.61–1.02)		0.19
Adjusted HR^a^	0.69 (0.50–0.95)		1.01 (0.77–1.33)		0.85 (0.66–1.10)		
Recurrent HF hospitalizations or CV death							
No. of events	112	185	199	224	255	329	
Rate per 100 patient‐years (95% CI)	9.1 (7.5–10.9)	15.8 (13.7–18.3)	17.1 (14.9–19.7)	18.9 (16.6–21.6)	24.0 (21.2–27.1)	30.6 (27.4–34.0)	
Rate ratio	0.57 (0.42–0.79)		0.90 (0.69–1.16)		0.79 (0.63–0.98)		0.10
Adjusted rate ratio^a^	0.56 (0.40–0.77)		0.90 (0.69–1.17)		0.84 (0.67–1.04)		
Significant worsening in KCCQ‐TSS (≥5) at 8 months							
Proportion ± SE (%)	23.8 ± 1.6	30.6 ± 1.8	27.8 ± 1.7	32.6 ± 1.7	24.7 ± 1.7	35.4 ± 1.8	
OR (95% CI)	0.85 (0.75–0.96)		0.90 (0.81–1.01)		0.77 (0.68–0.87)		0.47
Significant improvement in KCCQ‐TSS (≥5) at 8 months							
Proportion ± SE (%)	59.2 ± 1.9	53.2 ± 1.9	56.2 ± 1.8	50.5 ± 1.9	58.9 ± 1.9	49.0 + 1.9	
OR (95% CI)	1.13 (1.01–1.25)		1.11 (1.00–1.23)		1.22 (1.10–1.37)		0.50

CI, confidence interval; CV, cardiovascular; HF, heart failure; HR, hazard ratio; KCCQ‐TSS, Kansas City Cardiomyopathy Questionnaire total symptom score; OR, odds ratio; SE, standard error.

^a^ Adjusted for history of HF hospitalization, age, sex, race, region, systolic blood pressure, heart rate, left ventricular ejection fraction, estimated glomerular filtration rate, N‐terminal pro‐B‐type natriuretic peptide (log‐transformed), haemoglobin, New York Heart Association class, hypertension, stroke, prior myocardial infarction, atrial fibrillation, ischaemic aetiology.

The proportion of patients with a 5‐point or more decrease in KCCQ‐TSS (worsening) was smaller in those randomized to dapagliflozin, and the proportion of patients with a 5‐point or more increase in KCCQ‐TSS score (improvement) was higher in those randomized to dapagliflozin, irrespective of baseline bilirubin tertile (*Table* *3*).

There was no significant interaction between ALP, AST or ALT and randomized treatment on the occurrence of the primary outcome with the liver tests being modelled as continuous variables (online supplementary *Figure* *S4*).

### Effect of dapagliflozin on liver tests

End‐of‐study visit samples were spread as follows: 13.5% at 12 months, 25.9% at 16 months, 33.5% at 20 months, 23.9% at 24 months, and 3.2% at 28 months. Although this analysis suggested a small increase in bilirubin at the end‐of‐study visit in patients assigned to dapagliflozin (*Table* *4*), the supplementary analysis using additional results from unplanned samples showed no change in bilirubin or any other liver tests with dapagliflozin (online supplementary *Table* *S4*). There was no difference in change in liver tests when diabetic patients were analysed separately (data not shown).

**Table 4:** Change in liver tests at end‐of‐study visit by comparison of geometric mean ratios and by least squared mean regression

	*n*	Comparison of geometric means				Least squared mean regression	
		Baseline	Study end				
		Geometric mean (95% CI)	Geometric mean (95% CI)	Ratio: follow up/baseline geometric mean (95% CI)	Ratio: dapagliflozin/placebo	Mean change	Between treatment difference (dapagliflozin vs. placebo)
Bilirubin							
Dapagliflozin	1875	9.9 (9.7–10.2)	10.0 (9.8–10.3)	1.01 (0.99–1.03)	1.04 (1.01–1.07), *p* = 0.002	0.1 (−0.2, 0.3)	0.4 (0.0, 0.8), *p* = 0.05
Placebo	1819	9.9 (9.6–10.1)	9.6 (9.4–9.8)	0.97 (0.95–0.99)		−0.2 (−0.5, 0.1)	
ALP							
Dapagliflozin	1893	76.5 (75.3–77.6)	74.3 (73.2–75.5)	0.97 (0.96–0.98)	1.01 (1.00–1.03), *p* = 0.11	−2.0 (−3.3, −0.7)	0.7 (−1.1, 2.4), *p* = 0.44
Placebo	1824	76.7 (75.5–78.0)	73.6 (72.4–74.8)	0.96 (0.95–0.97)		−3.0 (−4.4, −1.6)	
ALT							
Dapagliflozin	1839	18.1 (17.7–18.5)	17.4 (17.0–17.8)	0.96 (0.94–0.98)	1.02 (0.99–1.05), *p* = 0.14	−0.5 (−1.1,0.2)	−1.2 (−4.4, 2.0), *p* = 0.45
Placebo	1768	18.7 (18.3–19.1)	17.3 (16.9–17.7)	0.93 (0.91–0.95)		0.4 (−2.8, 3.6)	
AST							
Dapagliflozin	1867	21.2 (20.8–21.5)	20.3 (19.9–20.6)	0.96 (0.94–0.97)	1.00 (0.98–1.02), *p* = 0.89	−0.7 (−1.3, −0.1)	−2.4 (−6.8, 2.0), *p* = 0.29
Placebo	1809	21.6 (21.3–22.0)	20.4 (20.1–20.8)	0.95 (0.93–0.96)		1.5 (−3.0, 6.0)	

ALP, alkaline phosphatase; ALT, alanine aminotransferase; AST, aspartate aminotransferase; CI, confidence interval.

### Safety and adverse events

Each of the adverse events of interest was uncommon. A similar proportion of patients experienced adverse events across bilirubin tertiles (online supplementary *Table* *S5*). The rate of adverse events did not differ notably between patients assigned to placebo or dapagliflozin, in any bilirubin tertile (online supplementary *Table* *S5*).

## Discussion

In a contemporary, well‐treated ambulatory cohort of patients with HFrEF, most of whom had mild symptoms, the prevalence of abnormal liver tests was low (ranging from 8% to 15% for the various liver tests measured), although patients with significant hepatic disease were not enrolled in DAPA‐HF. Bilirubin was the most frequently elevated liver test, and it remained an independent predictor of outcomes, despite adjustment for other prognostic variables, including NT‐proBNP and high‐sensitivity troponin T, a finding we believe has not been reported before. ALP was also independently predictive of outcome. The benefit of dapagliflozin was consistent across the range of bilirubin concentrations measured at baseline.

The proportion of patients in DAPA‐HF in which bilirubin was elevated (14.7%) was similar to that observed in PARADIGM‐HF (11.6%) and in the patients with reduced ejection fraction (15.8%) recruited approximately 20 years ago in CHARM when background therapy was markedly different.^10^, ^11^ In all three studies, bilirubin was associated with both the composite outcome of CV death or worsening HF and all‐cause mortality in predictive models including an array of clinical and routinely measured biochemical variables. In PARADIGM‐HF, bilirubin was an independent predictor of each outcome in multivariable models including these variables and NT‐proBNP, which is the single most powerful predictor of outcomes in HFrEF (NT‐proBNP was not measured in CHARM).^11^, ^24^ High‐sensitivity troponin has emerged as one of the few additional biomarkers to consistently add prognostic information when added to NT‐proBNP.^12^, ^25^, ^26^ In DAPA‐HF we tested whether bilirubin retained its independent predictive value even in models containing both NT‐proBNP and troponin, in addition to clinical variables. We found that bilirubin provided incremental prognostic information even when added to these other biomarkers and the fully aHR for each outcome related to bilirubin level was not attenuated to any significant extent compared to the unadjusted hazard ratio.

The interesting question is what aspect of HF pathophysiology is measured by bilirubin? Bilirubin is associated with high central venous/right atrial pressure in patients with HF and this finding raises the possibility that bilirubin provides different information about central haemodynamics than NT‐proBNP, which may be more reflective of left‐ than right‐sided pressures.^7^, ^8^, ^9^ Because bilirubin is associated with high central venous/right atrial pressure, we anticipated that dapagliflozin would reduce bilirubin as SGLT2 inhibitors have a diuretic action that might alleviate hepatic congestion.^15^, ^16^, ^17^ However, we did not find this, possibly because the diuretic action of SGLT2 inhibitors is short‐lived and might not have led to a sustained decrease in bilirubin (there were small numbers of bilirubin measurements before 12 months after randomization, therefore early change could not be assessed).^15^, ^16^, ^17^ Moreover, trials examining haemodynamic measurements have reported inconsistent findings. In a recent placebo‐controlled invasive haemodynamic study, 3 months of treatment with empagliflozin did not reduce right‐sided pressures.^27^ Conversely, in a trial in patients with an implanted pulmonary artery pressure sensor, empagliflozin significantly reduced pulmonary artery end‐diastolic pressure after 1 week, with a difference of 1.7 (95% CI 0.3–3.2) mmHg, compared with placebo (*p* = 0.02), by 12 weeks.^28^

This lack of effect on bilirubin contrasts with the observation that sacubitril/valsartan, when compared with enalapril, did reduce bilirubin significantly in PARADIGM‐HF.^11^ It is not clear why these two trials differed in this respect. Although there is no randomized controlled trial of the central haemodynamic effects of sacubitril/valsartan, this agent may have greater effects on preload and afterload than SGLT2 inhibitors as indirectly suggested by the much larger reduction in NT‐proBNP with sacubitril/valsartan compared to SGLT2 inhibitors.^29^, ^30^, ^31^, ^32^

Transaminase levels are not increased as often as bilirubin in patients with HF and transaminase levels may reflect a decrease in cardiac index and liver blood flow more than the elevation of central venous pressure.^7^, ^8^, ^9^ In DAPA‐HF dapagliflozin treatment was not associated with a reduction in transaminases. By contrast, sacubitril/valsartan did reduce AST and ALT in PARADIGM‐HF, suggesting either a specific effect of neprilysin inhibition on transaminase activity or, more likely, a greater effect of sacubitril/valsartan on preload and afterload, compared with an SGLT2 inhibitor.^11^ Consistent with this hypothesis, one recent study showed that the improvement in cardiac index with intravenous vasodilator and inotropic therapy in patients with advanced HF was maintained following a switch to sacubitril/valsartan (although this was not a controlled trial).^33^ SGLT2 inhibitors do decrease transaminases in patients with type 2 diabetes, but this is not thought to be haemodynamically mediated and, instead, probably reflects a reduction in visceral fat, accumulation of which is not thought to be a feature of HFrEF.^18^, ^19^, ^20^

Baseline bilirubin level did not modify the effect of dapagliflozin, as was also observed with sacubitril/valsartan (but was not examined with candesartan). This is important because these treatments are beneficial even in patients at high risk related to elevated bilirubin levels and the absolute benefits in such patients are large.

### Study limitations

This was not a pre‐specified analysis. Inclusion and exclusion criteria applied may have limited the generalizability of our findings. Specifically, patients with an AST or ALT more than three times the upper limit or normal (or bilirubin greater than twice the upper limit or normal) were excluded. We did not collect data on history of liver disease or alcohol intake. Other measures reflecting hepatic function, including albumin, platelet count and international normalized ratio, were not carried out in DAPA‐HF. We did not collect information on right‐sided filling pressures or right ventricular function. Regional variation in prevalence of sub‐hepatitis may account for some of the variation in abnormal liver tests, as patients were not screened for viral hepatitis.^34^ Scheduled sampling of liver tests occurred only at baseline and the end‐of‐study visit (between 12 and 28 months after randomization). Including only end‐of‐study visits may have introduced survivor bias and our supplementary analysis, including unscheduled visits, may not be a representative sample as there was an indication for additional investigation.

## Conclusion

Baseline bilirubin concentration was an independent predictor of worse outcomes but did not modify the benefits of dapagliflozin on morbidity and mortality in HFrEF.

### Funding

The DAPA‐HF trial was funded by AstraZeneca. C.A. and J.J.V.M. are supported by a British Heart Foundation Centre of Research Excellence Grant RE/18/6/34217.

**Conflict of interest**: The UMCG, which employs Dr. de Boer has received research grants and/or fees from AstraZeneca, Abbott, Boehringer Ingelheim, Cardior Pharmaceuticals Gmbh, Ionis Pharmaceuticals, Inc., Novo Nordisk, and Roche. R.A.d.B. received speaker fees from Abbott, AstraZeneca, Bayer, Novartis, and Roche. M.D. reports personal fees from AstraZeneca. J.D. has received personal and institutional research support for DELIVER from AstraZeneca. S.E.I. has received honoraria for advisory work and/or clinical trial leadership from AstraZeneca, Boehringer Ingelheim, Novo Nordisk, Merck, Pfizer, Lexicon, Esperion, vTv Therapeutics, and Abbott; has delivered lectures sponsored by AstraZeneca and Boehringer Ingelheim. L.K. reports speakers honoraria from Novo Nordisk, Novartis, AstraZeneca and Boehringer Ingelheim; support from AstraZeneca; and personal fees from Novartis and Bristol Myers Squibb as a speaker. M.N.K. reports payment to his institution for participation in DAPA‐HF; has received grant payment to his institution from Boehringer Ingelheim; has received personal fees or fees to his institution, or both, for consultancy from Amgen, Applied Therapeutics, AstraZeneca, Bayer, Boehringer Ingelheim, Eli Lilly, Esperion Therapeutics, Janssen, Merck, Novo Nordisk, Sanofi, and Vifor Pharma; has received personal honoraria and honoraria to his institution for lectures from AstraZeneca, Boeringer Ingelheim, and Novo Nordisk; has received personal honoraria and honoraria to his institution from Amgen, Applied Therapeutics, AstraZeneca, Bayer, Boehringer Ingelheim, Eli Lilly, Janssen, Merck, Novo Nordisk, Sanofi, and Vifor Pharma for participation on DSMB for advisory boards; and has received study drug for a clinical trial from AstraZeneca and Boehringer Ingelheim. C.E.A.L. reports personal fees from AstraZeneca during the conduct of the study and personal fees from AstraZeneca, Novartis, and Pfizer outside the submitted work. F.A.M. reports personal fees from AstraZeneca. P.P. reports personal fees for consultancy and speakers bureau from AstraZeneca, Boehringer Ingelheim, Vifor Pharma, Servier, Bayer, Bristol Myers Squibb, Respocardia, Berlin‐Chemie, Cibiem, Novartis and RenalGuard; other support for participation in clinical trials from Boehringer Ingelheim, Amgen, Vifor Pharma, Bayer, Bristol Myers Squibb, Cibiem, Novartis and RenalGuard; and research grants to his institution from Vifor Pharma. M.S.S. received an institutional research grant from AstraZeneca for DAPA‐HF; received institutional research grants from Abbott, Amgen, Anthos Therapeutics, Bayer, Daiichi‐Sankyo, Eisai, Intarcia, IONIS, The Medicines Company, MedImmune, Merck, Novartis, Pfizer, and Quark Pharmaceuticals; received consulting fees from Althera, Amgen, Anthos Therapeutics, AstraZeneca, Bristol Myers Squibb, CVS Caremark, DalCor, Dr Reddy's Laboratories, Fibrogen, IFM Therapeutics, Intarcia, MedImmune, Merck, Moderna, and Novo Nordisk; and is a member of the TIMI Study Group, which has also received institutional research grant support through Brigham and Women's Hospital from Regeneron, Roche, and Zora Biosciences. D.L., O.B., D.W.B., P.J.G., A.M.L. and M.S. are employees of AstraZeneca. S.D.S. received payment to his institution for participation in DAPA‐HF; received research grants from Actelion, Alnylam, Amgen, AstraZeneca, Bellerophon, Bayer, Bristol Myers Squibb, Celladon, Cytokinetics, Eidos, Gilead, GlaxoSmithKline, Ionis, Lilly, Mesoblast, MyoKardia, National Institutes of Health/NHLBI, Neurotronik, Novartis, NovoNordisk, Respicardia, Sanofi Pasteur, Theracos; and consulted for Abbott, Action, Akros, Alnylam, Amgen, Arena, AstraZeneca, Bayer, Boehringer Ingelheim, Bristol Myers Squibb, Cardior, Cardurion, Corvia, Cytokinetics, Daiichi‐Sankyo, GlaxoSmithKline, Lilly, Merck, Myokardia, Novartis, Roche, Theracos, Quantum Genomics, Cardurion, Janssen, Cardiac Dimensions, Tenaya, Sanofi‐Pasteur, Dinaqor, Tremeau, CellProThera, Moderna, American Regent and Sarepta. J.J.V.M. declares payments to his employer, Glasgow University, for his work on clinical trials, consulting and other activities: Alnylam, Amgen, AstraZeneca, Bayer, Boehringer Ingelheim, BMS, Cardurion, Cytokinetics, Dal‐Cor, GSK, Ionis, KBP Biosciences, Novartis, Pfizer, Theracos; personal lecture fees from Abbott, Alkem Metabolics, AstraZeneca, Eris Lifesciences, Hikma, Lupin, Sun Pharmaceuticals, Medscape/Heart.Org, ProAdWise Communications, S & L Solutions Event Management Inc, Radcliffe Cardiology, Servier, the Corpus, Translational Medical Academy, Web MD and (as Director) the Global Clinical Trial Partners Ltd (GCTP). P.S.J. reports payment to the University of Glasgow by AstraZeneca for his time working on the DAPA‐HF and DELIVER trials, from Novartis for work on the PARADIGM‐HF and PARAGON‐HF trials, and Novo Nordisk; reports speakers and advisory board fees from AstraZeneca, Boehringer Ingelheim, and Novartis; and reports research funding from Boehringer Ingelheim and Analog Devices. All other authors have nothing to disclose.

## Supporting information


**Appendix S1.** Supporting information.
