# Supplementary material for: Liver tests and outcomes in heart failure with reduced ejection fraction: findings from DAPA‐HF
Source: Eur J Heart Fail. 2022 Aug 22;24(10):1856–68. doi: 10.1002/ejhf.2649 (PMC9805158; doi:10.1002/ejhf.2649)
Supplement: Supplementary file 1 — Appendix S1. Supporting information. [file EJHF-24-1856-s001.docx]

**Supplemental Appendix**

[Supplementary Table 1 2](#_Toc109375101)

[Supplementary Table 2 3](#_Toc109375102)

[Supplementary Table 3. 5](#_Toc109375103)

[Supplementary Table 4 7](#_Toc109375104)

[Supplementary Table 5 10](#_Toc109375105)

[Supplementary Figure 1 11](#_Toc109375106)

[Supplementary Figure 2 12](#_Toc109375107)

[Supplementary Figure 3 13](#_Toc109375108)

[Supplementary Figure 4 14](#_Toc109375109)

Supplementary Table 1

Baseline characteristics associated with high bilirubin in a multivariable model

| NT-proBNP > median | 2.02 (95% CI 1.6-2.54) |
| --- | --- |
| Hb > median | 1.90 (95% CI 1.51-2.39) |
| History of AF | 1.65 (95% CI 1.32-2.05) |
| Female sex | 0.44 (95% CI 0.31-0.62) |
| NYHA class III/IV | 1.49 (95% CI 1.18-1.88) |
| AST > median | 1.61 (95% CI 1.29-2) |
| Pulse pressure > median | 0.66 (95% CI 0.53-0.82) |
| KCCQ-TSS > median | 0.73 (95% CI 0.58-0.91) |
| ALP > median | 1.26 (95% CI 1.01-1.57) |

Each candidate variable assessed in univariable logistic regression for odds of bilirubin greater than upper normal reference range at baseline. Continuous variables were dichotomized at the median population value. Candidate variables: age; sex; obesity; race; region; systolic BP; pulse pressure; heart rate; alkaline phosphatase; AST; eGFR; NT-proBNP; high sensitivity troponin-T; urea; HbA1c; hemoglobin; hematocrit; ischemic etiology; duration of HF; time from HF most recent HF hospitalization to randomization; LVEF; NYHA III/IV vs II; KCCQ-TSS; hypertension; TIIDM; AF; history of HF hospitalization; prior MI; stroke; COPD; smoking status; ACEI/ARB/ARNI; diuretic; digoxin; beta blocker; MRA; anticoagulant; antiplatelet; statin; amiodarone; ICD/CRT-D. AST and ALT were highly correlated (Pearson correlation coefficient 0.75 [p<0.001]) only one was entered into the model – AST selected as had a lower p value on univariable analysis. Hematocrit and hemoglobin were highly correlated (Pearson coefficient 0.82) therefore only hemoglobin was entered into the model. Variables with p value < 0.2 in univariable analysis were entered into a stepwise logistic regression model with a p value of 0.05 to be entered into the model.

# Supplementary Table 2

Hazard ratios/rate ratios for key study outcomes according to tertile of total bilirubin at baseline in male patients only

|  | **1^st^ Tertile**  **(*n* = 1090)** | **2^nd^ Tertile**  **(*n* = 1241)** | ***P*-value** | **3^rd^ Tertile**  **(*n* = 1284)** | ***P*-value** |
| --- | --- | --- | --- | --- | --- |
| **Primary endpoint – no. (%)** | 146 (13.4) | 229 (18.5) |  | 335 (26.1) |  |
| Event rate – no. of cases per 100 patient years (95% CI) | 9.3 (7.9-10.9) | 13.4 (11.7-15.2) |  | 16.7 (18.0-22.3) |  |
| Unadjusted hazard ratio (95% CI) | 1.00 (reference) | 1.46 (1.18-1.79) | <0.001 | 2.24 (.84-2.72) | <0.001 |
| Adjusted hazard ratio 1 (95% CI) | 1.00 (reference) | 1.25 (1.01-1.54) | 0.04 | 1.68 (1.36-2.07) | <0.001 |
| Adjusted hazard ratio 2 (95% CI) | 1.00 (reference) | 1.14 (0.87-1.49) | 0.33 | 1.67 (1.29-2.17) | <0.001 |
| **HF urgent visit or hospitalization – no. (%)** | 78 (7.2) | 140 (11.3) |  | 226 (17.6) |  |
| Event rate – no. of cases per 100 patient years (95% CI) | 5.0 (4.0-6.2) | 8.2 (6.9-9.6) |  | 13.5 (11.9-15.4) |  |
| Unadjusted hazard ratio (95% CI) | 1.00 (reference) | 1.67 (1.27-2.21) | <0.001 | 2.84 (2.20-3.68) | <0.001 |
| Adjusted hazard ratio 1 (95% CI) | 1.00 (reference) | 1.41 (1.06-1.87) | 0.02 | 2.16 (1.64-2.84) | <0.001 |
| Adjusted hazard ratio 2 (95% CI) | 1.00 (reference) | 1.27 (0.89-1.82) | 0.18 | 2.12 (1.51-2.97) | <0.001 |

| **Supplementary Table 2. (continued)** | | | | | |
| --- | --- | --- | --- | --- | --- |
|  | **1^st^ Tertile**  **(*n* = 1090)** | **2^nd^ Tertile**  **(*n* = 1241)** | ***P*-value** | **3^rd^ Tertile**  **(*n* = 1284)** | ***P*-value** |
| **Death from cardiovascular causes – no. (%)** | 88 (8.1) | 136 (11.0) |  | 188 (14.6) |  |
| Event rate – no. of cases per 100 patient years (95% CI) | 5.4 (4.4-6.7) | 7.6 (6.4-8.9) |  | 10.3 (8.9-11.9) |  |
| Unadjusted hazard ratio (95% CI) | 1.00 (reference) | 1.42 (1.08-1.85) | 0.01 | 1.96 (1.52-2.52) | <0.001 |
| Adjusted hazard ratio 1 (95% CI) | 1.00 (reference) | 1.25 (0.95-1.64) | 0.12 | 1.41 (1.07*1.86) | 0.01 |
| Adjusted hazard ratio 2 (95% CI) | 1.00 (reference) | 1.24 (0.88-1.76) | 0.22 | 1.42 (1.01-1.99) | 0.05 |
| **Death from any cause – no. (%)** | 108 (9.9) | 174 (14.0) |  | 213 (16.6) |  |
| Event rate – no. of cases per 100 patient years (95% CI) | 6.7 (5.5-8.0) | 9.7 (8.3-11.2) |  | 11.7 (10.2-13.3) |  |
| Unadjusted hazard ratio (95% CI) | 1.00 (reference) | 1.48 (1.16-1.88) | 0.001 | 1.80 (1.43-2.27) | <0.001 |
| Adjusted hazard ratio 1 (95% CI) | 1.00 (reference) | 1.30 (1.02-1.66) | 0.04 | 1.34 (1.05-1.72) | 0.02 |
| Adjusted hazard ratio 2 (95% CI) | 1.00 (reference) | 1.26 (0.93-1.72) | 0.14 | 1.32 (0.97-1.79) | 0.08 |

For the primary endpoint, HF urgent visit or hospitalization, CV death, and death from any cause, models are adjusted for previous hospitalization for HF and treatment allocation and stratified by diabetic status. For death from any cause, models are adjusted for treatment allocation and stratified according to diabetic status.

Adjusted model 1 includes additional adjustment for age, sex, race, region, systolic blood pressure, heart rate, LVEF, eGFR, NT-proBNP (log-transformed), hemoglobin, NYHA class, hypertension, previous stroke, previous MI, atrial fibrillation, and HF etiology. Adjusted model 2 has the same variables as model 1 with plus (log transformed) high sensitivity troponin T.

Abbreviations: eGFR, estimated glomerular filtration rate; HF, heart failure; LVEF, left ventricular ejection fraction; MI, myocardial infarction; NT-proBNP, N-terminal pro B-type natriuretic peptide, NYHA, New York heart association.

# Supplementary Table 3.

Risk of outcomes by liver function tests

|  | **Crude HR /RR(95% CI)** | | ***P*-value** | | **Adjusted HR/RR 1 (95% CI)** | | ***P*-value** | | **Adjusted HR/RR 2 (95% CI)** | | ***P*-value** | |  |
| --- | --- | --- | --- | --- | --- | --- | --- | --- | --- | --- | --- | --- | --- |
| **Primary endpoint** | | | | | | | | | | | | |  |
| **AST** | 1.14 (0.94-1.37) | | 0.18 | | 1.12 (0.92-1.36) | | 0.25 | | 1.02 (0.80-1.30) | | 0.90 | |  |
| **ALT** | 0.98 (0.85-1.12) | | 0.75 | | 1.08 (0.93-0.96) | | 0.30 | | 0.99 (0.83-1.18) | | 0.91 | |  |
| **ALP** | 1.89 (1.58-2.26) | | <0.001 | | 1.35 (1.13-1.62) | | 0.001 | | 1.25 (1.00-1.58) | | 0.05 | |  |
| **Total bilirubin** | 1.95 (1.72-2.21) | | <0.001 | | 1.54 (1.34-1.76) | | <0.001 | | 1.66 (1.39-1.98) | | <0.001 | |  |
| **Hospitalization or urgent visit for HF** | | | | | | | | | | | | |  |
| **AST** | 1.10 (0.86-1.39) | | 0.45 | | 1.05 (0.85-1.34) | | 0.71 | | 0.94 (0.69-1.28) | | 0.69 | |  |
| **ALT** | 0.96 (0.80-1.14) | | 0.62 | | 1.07 (0.61-0.86) | | 0.48 | | 0.98 (0.79-1.23) | | 0.89 | |  |
| **ALP** | 1.78 (1.42-2.22) | | <0.001 | | 1.26 (1.00-1.59) | | 0.05 | | 1.21 (0.91-1.61) | | 0.18 | |  |
| **Total bilirubin** | 2.22 (1.89-2.59) | | <0.001 | | 1.76 (1.49-2.09) | | <0.001 | | 1.94 (1.56-2.40) | | <0.001 | |  |
| **Death from cardiovascular causes** | | | | | | | | | | | | |  |
| **AST** | 1.13 (0.88-1.45) | | 0.35 | | 1.12 (0.87-1.45) | | 0.38 | | 0.96 (0.0-1.32) | | 0.81 | |  |
| **ALT** | 0.95 (0.78-1.14) | | 0.56 | | 1.02 (0.84-1.23) | | 0.85 | | 0.89 (0.7-1.13) | | 0.34 | |  |
| **ALP** | 1.99 (1.58-2.52) | | <0.001 | | 1.30 (1.03-1.66) | | 0.03 | | 1.24 (0.92-1.68) | | 0.16 | |  |
| **Total bilirubin** | 1.88 (1.60-2.23) | | <0.001 | | 1.39 (1.16-1.67) | | <0.001 | | 1.46 (1.16-1.85) | | 0.001 | |  |
| **Death from any cause** | | | | | | | | | | | | | |
| **AST** | | 1.03 (0.82-1.30) | | 0.81 | | 1.06 (0.84-1.33) | | 0.07 | | 0.91 (0.67-1.22) | | 0.51 | |
| **ALT** | | 0.84 (0.71-1.00) | | 0.05 | | 0.94 (0.79-1.11) | | 0.46 | | 0.82 (0.66-1.02) | | 0.08 | |
| **ALP** | | 2.12 (1.72-2.62) | | <0.001 | | 1.43 (1.16-1.78) | | 0.001 | | 1.38 (1.05-1.82) | | 0.02 | |
| **Total bilirubin** | | 1.69 (1.45-1.96) | | 0.03 | | 1.31 (1.11-1.54) | | 0.002 | | 1.33 (1.07-1.64) | | 0.01 | |
| **Recurrent HF hospitalizations or cardiovascular death** | | | | | | | | | | | | | |
| **AST** | | 1.08 (0.92-1.26) | | 0.36 | | 1.04 (0.89-1.22) | | 0.59 | | 0.92 (0.76-1.12) | | 0.42 | |
| **ALT** | | 0.94 (0.84-1.05) | | 0.28 | | 1.04 (0.93-1.17) | | 0.52 | | 0.96 (0.83-1.11) | | 0.58 | |
| **ALP** | | 1.81 (1.57-2.10) | | <0.001 | | 1.23 (1.06-1.43) | | 0.01 | | 1.17 (0.97-1.40) | | 0.10 | |
| **Total bilirubin** | | 2.07 (1.87-2.29) | | <0.001 | | 1.58 (1.41-1.77) | | <0.001 | | 1.67 (1.45-1.93) | | <0.001 | |

All liver function tests have been log transformed. HR are given for one unit increase in the log-transformed LFT.

For the primary endpoint, HF urgent visit or hospitalization, CV death, and death from any cause, models are adjusted for previous hospitalization for HF and treatment allocation and stratified by diabetic status. For death from any cause, models are adjusted for treatment allocation and stratified according to diabetic status.

Adjusted model 1 includes additional adjustment for age, sex, race, region, systolic blood pressure, heart rate, LVEF, eGFR, NT-proBNP (log-transformed), haemoglobin, NYHA class, hypertension, previous stroke, previous MI, atrial fibrillation, and HF etiology. Adjusted model 2 has the same variables as model 1 with plus high sensitivity troponin T (log transformed).

Abbreviations: ALP, alkaline phosphatase; ALT, alanine transaminase; AST, aspartate transaminase; CI, confidence interval; eGFR, estimated glomerular filtration rate; HF, heart failure; HR, hazard ratio; LVEF, left ventricular ejection fraction; MI, myocardial infarction; NT-proBNP, N-terminal pro B-type natriuretic peptide; NYHA, New York heart association; RR, rate ratio

# Supplementary Table 4

Change in liver function tests at 12/16 months and 20/24 months of follow up

1. Least squared mean regression

|  | **12/16m*** | | **20/24m†** | |
| --- | --- | --- | --- | --- |
|  | Placebo | Dapagliflozin | Placebo | Dapagliflozin |
| **Bilirubin (µmol/L)** |  |  |  |  |
| N | 788 | 803 | 1058 | 1076 |
| Mean change | -0.3 (-0.9, 0.3) | 0.1 (-0.3, 0.5) | -0.3 (-0.6, 0.0) | 0.1 (-0.3, 0.4) |
| Between treatment difference (dapagliflozin vs placebo) | 0.5 (-0.2,1.2), p = 0.16 | | 0.3 (-0.1, 0.7), p = 0.12 | |
| **ALP (IU/L)** |  |  |  |  |
| N | 792 | 804 | 1062 | 1092 |
| Mean change | -0.9 (-3.2, 1.3) | -2.1 (-3.9, -0.3) | -4.4 (-6.2, -2.7) | -2.2 (-4.0, -0.4) |
| Between treatment difference (dapagliflozin vs placebo) | -0.9 (-3.6, 1.8), p = 0.50 | | 1.71 (-0.5, 4.0), p = 0.14 | |
| **ALT (IU/L)** |  |  |  |  |
| N | 785 | 800 | 1052 | 1071 |
| Mean change | 3.2 (-4.1, 10.6) | -0.8 (-1.9, 0.3) | -1.7 (-2.5, -0.9) | -0.3 (-1.1, 0.5) |
| Between treatment difference (dapagliflozin vs placebo) | -4.4 (-11.7, 2.9), p = 0.24 | | 1.1 (0.1, 2.1), p = 0.03 | |
| **AST (IU/L)** |  |  |  |  |
| N | 763 | 786 | 1036 | 1056 |
| Mean change | 4.9 (-5.5, 15.3) | -1.0 (-1.9, -0.1) | -1.1 (-1.8, -0.5) | -0.5 (-1.3, 0.2) |
| Between treatment difference (dapagliflozin vs placebo) | -6.1 (-16.4, 4.1), p = 0.24 | | 0.4 (-0.4, 1.3), p = 0.31 | |

* Either a recording at 12m OR 16m or if both present an average of the two recorded values

† Either a recording at 20m OR 24m or if both present an average of the two recorded values

1. Geometric mean ratios

|  | Baseline § | 12/16 months * | | | | 20/24 months † | | |
| --- | --- | --- | --- | --- | --- | --- | --- | --- |
|  | Geometric mean  (95% CI) | Geometric mean  (95% CI) | Ratio:  Follow up/baseline geometric mean (95% CI) | Ratio:  Dapagliflozin  /Placebo | | Geometric mean  (95% CI) | Ratio:  Follow up/baseline geometric mean (95% CI) | Ratio:  Dapagliflozin  /Placebo |
| Bilirubin |  |  |  | |  |  |  |  |
| Dapagliflozin | 10.1  (9.9-10.3) | 10.3  (9.9-10.7) | 1.01  (0.98-1.04) | | 1.07  (1.02-1.12)  P = 0.001 | 9.8  (9.5-10.1) | 1.00  (0.97-1.03) | 1.02  (0.99-1.05)  P = 0.25 |
| Placebo | 10.1  (9.9-10.3) | 9.5  (9.1-9.8) | 0.95  (0.92-0.98) | |  | 9.6  (9.3-9.9) | 0.98  (0.95-1.00) |  |
| ALP |  |  |  | |  |  |  |  |
| Dapagliflozin | 77.8  (76.7-78.9) | 75.2  (73.4-77.0) | 0.98  (0.96-0.99) | | 1.00  (0.98-1.02)  P = 0.94 | 74.0  (72.6-75.5) | 0.97  (0.95-0.98) | 1.02  (1.00-1.04)  P = 0.05 |
| Placebo | 78.1  (77.0-79.2) | 75.2  (73.3-77.0) | 0.98  (0.96-0.99) | |  | 72.7  (71.1-74.2) | 0.95  (0.93-0.96) |  |
| ALT |  |  |  | |  |  |  |  |
| Dapagliflozin | 18.0  (17.7-18.4) | 17.0  (16.4-17.6) | 0.94  (0.91-0.98) | | 0.97  (0.93-1.02)  P = 0.22 | 17.7  (17.2-18.2) | 0.97  (0.94-1.00) | 1.05  (1.01-1.09)  P = 0.01 |
| Placebo | 18.4  (18.1-18.8) | 17.7  (17.1-18.4) | 0.95  (0.92-0.99) | |  | 17.0  (16.6-17.5) | 0.92  (0.89-0.94) |  |
| AST |  |  |  | |  |  |  |  |
| Dapagliflozin | 21.2  (20.9-21.5) | 19.9  (19.4-20.4) | 0.95  (0.92-0.97) | | 0.97  (0.94-1.00)  P = 0.10 | 20.6  (20.1-21.1) | 0.96  (0.94-0.98) | 1.01  (0.99-1.04)  P = 0.31 |
| Placebo | 21.5  (21.1-21.8) | 20.7  (20.1-21.3) | 0.97  (0.94-0.99) | |  | 20.4  (20.0-20.9) | 0.94  (0.92-0.96) |  |

§ Baseline includes all patients with baseline value, regardless of whether they have follow up values

* Either a recording at 12m OR 16m or if both present an average of the two recorded values

† Either a recording at 20m OR 24m or if both present an average of the two recorded values

Abbreviations: ALP, alkaline phosphatase; ALT, alanine aminotransferase; AST, aspartate aminotransferase

# Supplementary Table 5

Adverse events related to randomized therapy, according to tertile of total bilirubin at baseline

|  | **1^st^ tertile** | | **2^nd^ tertile** | | **3^rd^ tertile** | | *P for interaction*^a^ |
| --- | --- | --- | --- | --- | --- | --- | --- |
|  | **Placebo**  **(n=794)** | **Dapagliflozin**  **(n=818)** | **Placebo**  **(n=804)** | **Dapagliflozin**  **(n=798)** | **Placebo**  **(n=765)** | **Dapagliflozin**  **(n=741)** |  |
| **Any discontinuation – no. (%)** | 85 (10.7%) | 95 (11.6%) | 74 ( 9.2%) | 78 ( 9.8%) | 99 (12.9%) | 74 (10.0%) | 0.18 |
| **Discontinuation due to adverse event – no. (%)** | 35 ( 4.4%) | 38 ( 4.6%) | 38 ( 4.7%) | 36 ( 4.5%) | 43 ( 5.6%) | 37 ( 5.0%) | 0.86 |
| **Adverse events – no. (%)** |  |  |  |  |  |  |  |
| Volume depletion | 49 ( 6.2%) | 62 ( 7.6%) | 61 ( 7.6%) | 54 ( 6.8%) | 52 ( 6.8%) | 61 ( 8.2%) | 0.37 |
| Renal | 51 ( 6.4%) | 47 ( 5.7%) | 65 ( 8.1%) | 66 ( 8.3%) | 54 ( 7.1%) | 40 ( 5.4%) | 0.56 |
| Fracture | 15 ( 1.9%) | 16 ( 2.0%) | 20 ( 2.5%) | 18 ( 2.3%) | 14 ( 1.8%) | 14 ( 1.9%) | 0.95 |
| Amputation | 6 ( 0.8%) | 5 ( 0.6%) | 4 ( 0.5%) | 6 ( 0.8%) | 2 ( 0.3%) | 2 ( 0.3%) | 0.71 |
| Major hypoglycaemia | 2 ( 0.3%) | 3 ( 0.4%) | 2 ( 0.2%) | 1 ( 0.1%) | 0 ( 0.0%) | 0 ( 0.0%) | 0.46 |

^a^Interaction between bilirubin tertile and effect of randomized treatment.

The safety analysis included only patients who took at least one dose of randomized treatment

# Supplementary Figure 1

Distribution of serum bilirubin at baseline in DAPA-HF.

|  |  |
| --- | --- |

# Supplementary Figure 2

Relationship between each liver test and the main trial outcomes.

|  | **Primary endpoint** | **Worsening HF event** | **CV death** | **All cause death** |
| --- | --- | --- | --- | --- |
| **Alkaline phosphatase** | **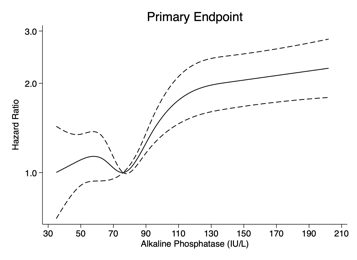** | **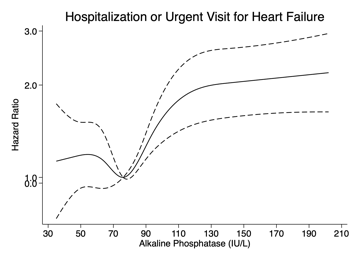** | **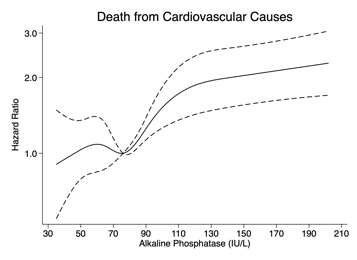** | **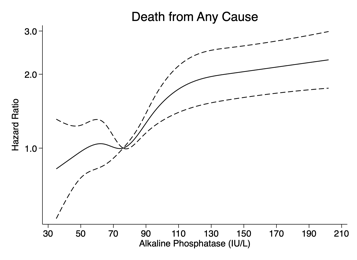** |
| **ALT** | **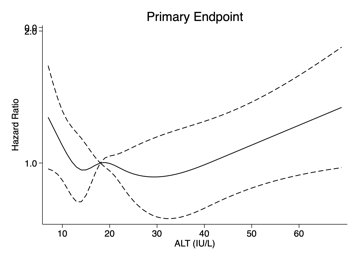** | **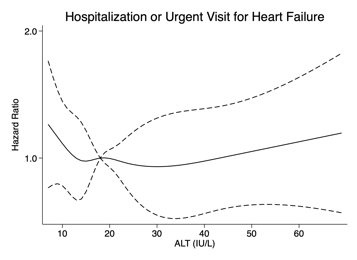** | **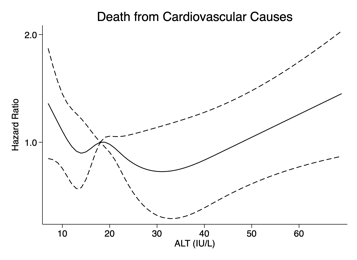** | **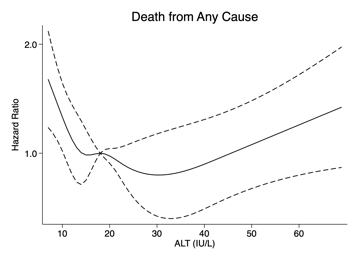** |
| **AST** | **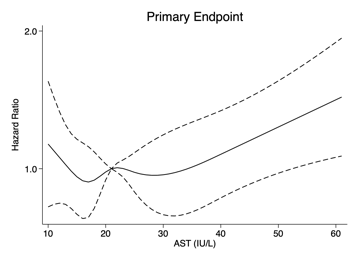** | **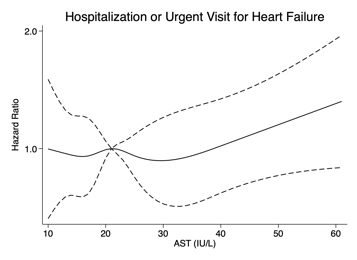** | **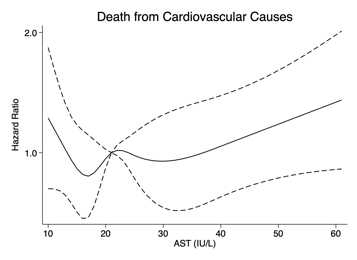** | **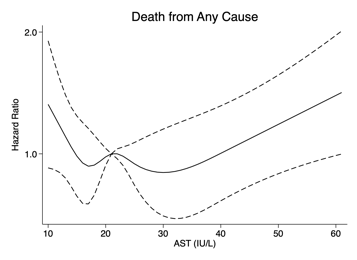** |

Relationship between continuous value of each liver test and outcome modelled as restricted cubic splines with 5 knots at default positions, adjusted for randomized treatment. Reference value is the population median, graphs are truncated at 1^st^ and 99^th^ centile of distribution.

# Supplementary Figure 3

Effect of randomized treatment over baseline bilirubin as a continuous variable on the main study endpoints.

| **A**  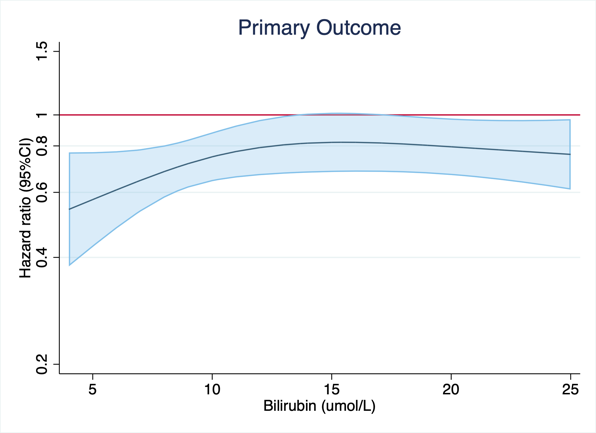  p for interaction = 0.20 | **B**  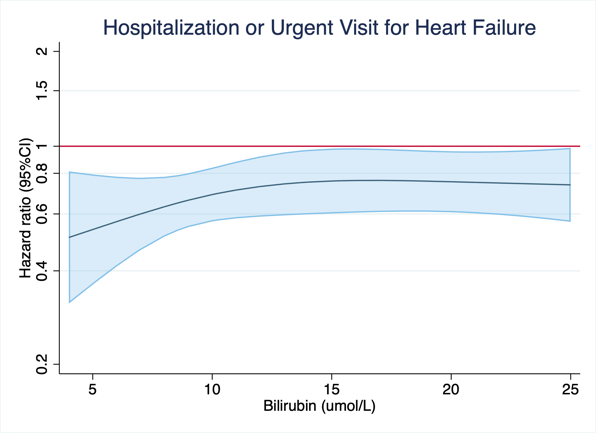  p for interaction = 0.40 |
| --- | --- |
| **C**  **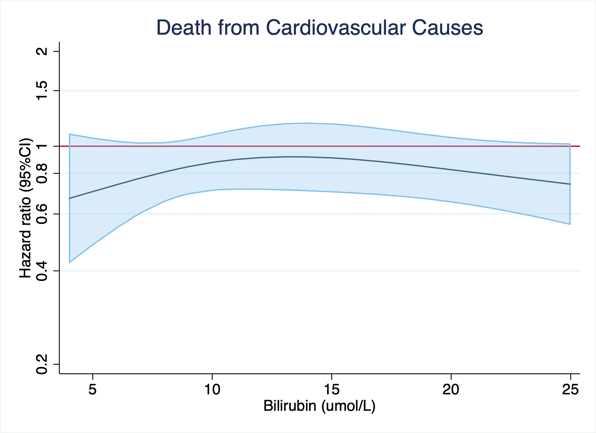**  p for interaction = 0.38 | **D**  **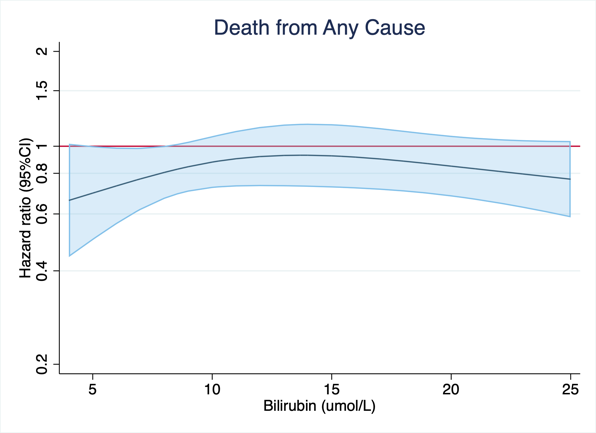**  p for interaction = 0.34 |

Effect of randomized treatment across baseline bilirubin as a continuous variable modelled using restricted cubic splines for the primary composite endpoint (A), hospitalization or urgent visit for heart failure (B), death from cardiovascular causes (C), or death from any cause (D). Interaction p-value is given for full range of bilirubin, graphic drawn for values between 5^th^ and 95^th^ centile

# Supplementary Figure 4

Effect of randomized treatment over other liver tests as continuous variables on the primary study endpoint

| A  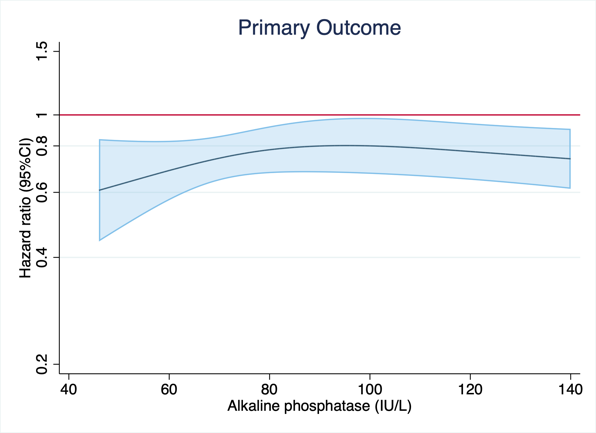  P for interaction = 0.16 | B  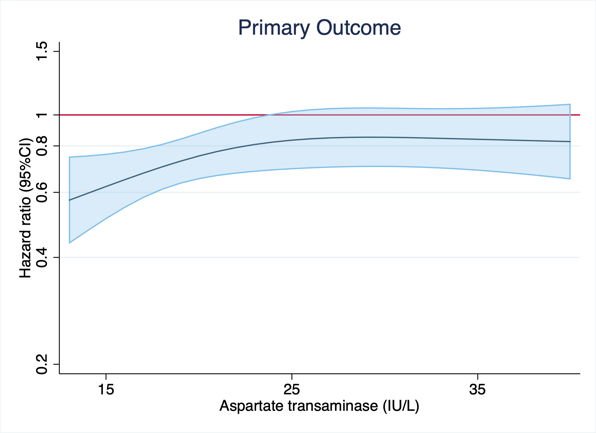  P for interaction = 0.12 | C  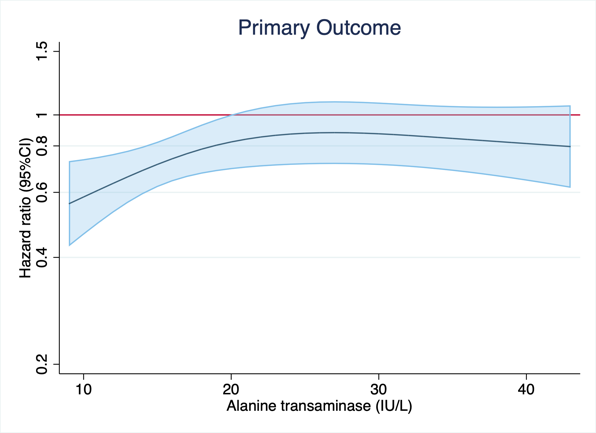  P for interaction = 0.07 |
| --- | --- | --- |

Effect of randomized treatment by baseline liver test [A, alkaline phosphatase; B, aspartate transaminase; C, alanine transaminase] as a continuous variable modelled using restricted cubic splines for the primary composite endpoint. Interaction p-value is given for full range of liver test, graphic drawn for values between 5^th^ and 95^th^ centile.
